# Effectiveness of ChatGPT, Google Gemini, and Microsoft Copilot in Answering Thai Drug Information Queries: Cross-Sectional Study

**DOI:** 10.2196/79751

**Published:** 2025-12-15

**Authors:** Suphannika Pornwattanakavee, Nattawut Leelakanok, Teerarat Todsarot, Gabrielle Angele Tatta Guinto, Ratchanon Takun, Assadawut Sumativit, Marisa Senngam

**Affiliations:** 1 Division of Clinical Pharmacy Faculty of Pharmaceutical Sciences Burapha University Chonburi Thailand

**Keywords:** artificial intelligence, chatbot, drug information, medication information, accuracy, correctness, completeness, risk assessment, ChatGPT-4o, Google Gemini, Microsoft Copilot

## Abstract

**Background:**

ChatGPT-4o, Google Gemini, and Microsoft Copilot have shown potential in generating health care–related information. However, their accuracy, completeness, and safety for providing drug-related information in Thai contexts remain underexplored.

**Objective:**

This study aims to evaluate the performance of artificial intelligence (AI) systems in responding to drug-related questions in Thai.

**Methods:**

An analytical cross-sectional study was conducted using 76 public drug-related questions compiled from medical databases and social media between November 1, 2019, and December 31, 2024. All questions were categorized into 19 distinct categories, each comprising 4 questions. ChatGPT-4o, Google Gemini, and Microsoft Copilot were queried in a single session on March 1, 2025, by using input in Thai. All responses were evaluated for correctness, completeness, risk, and reproducibility independently by clinical pharmacists using standardized evaluation criteria.

**Results:**

All 3 AI models provided generally complete responses (*P*=.08). ChatGPT-4o yielded the highest proportion of fully correct responses (*P*=.08). The overall risk levels of high-risk answers were not significantly different (*P*=.12). Response correctness was influenced by the category of the drug-related questions (*P*=.002) but not completeness (*P*=.23). The correctness of Google Gemini and Microsoft Copilot was higher than that of ChatGPT for pharmacology queries. The type of questions also statistically significantly affected the risk level of the answers (*P*=.04). In particular, the pregnancy and lactation category had the highest high-risk response rate (1/76, 1% per system). All 3 AI models demonstrated consistent response patterns when the same questions were re-queried after 1, 7, and 14 days.

**Conclusions:**

The evaluated AI chatbots were able to answer the queries with generally complete content; however, we found limited accuracy and occasional high-risk errors in responding to drug-related questions in Thai. All models exhibited good reproducibility.

## Introduction

Large language models such as ChatGPT, Google Gemini, and Microsoft Copilot have demonstrated potential in generating human-like responses, including for medication-related queries [1]. Traditionally, the public has relied on health care professionals for drug information; however, the growing accessibility of the internet and social media platforms has shifted this behavior. Generative artificial intelligence (AI) technologies are now playing a crucial role in facilitating prompt and efficient responses to health-related questions. A recent survey showed that more than 70% of the people expressed willingness to use AI applications such as ChatGPT for self-diagnosis and health-related queries [2]. Notably, GPT-4.0 has demonstrated marked improvements in both accuracy and safety over its earlier versions, including GPT-3.0 and 3.5 [3]. Because these AIs are primarily trained in English, they have reduced performance in non-English pharmaceutical contexts, such as in Chinese, Japanese, and Turkish [4-7]. In addition, the performance of generative AI varies across languages due to differences in the linguistic structure, question complexity, domain-specific content, cultural factors, and regulatory frameworks [8-10].

Launched in May 2024, ChatGPT-4o replaced ChatGPT-4.0. Advanced natural language processing features, including real-time interaction, multimodal input handling, and enhanced multilingual comprehension, provided by ChatGPT-4o, offer strong potential for communication enhancement between users and large language models in medical contexts [11]. Recent evaluations have shown that ChatGPT-4o significantly outperformed GPT-4.0 and GPT-3.5 in multiple-choice questions from the United States Medical Licensing Examination, with superior accuracy across overall, preclinical, and clinical domains [12]. A network meta-analysis confirmed that ChatGPT-4o showed better performance than GPT-4.0, Microsoft Copilot, and Google Gemini [13]. Despite these promising outcomes in structured assessments, there is insufficient evidence regarding the performance of ChatGPT-4o in open-ended medical and drug-related questions, especially in non-English contexts.

In Thailand, unique linguistic and contextual features, including the use of local trade names, Thai-language medical terminology, and region-specific clinical practices, may further affect the performance of large language models. ChatGPT-4o, Google Gemini, and Microsoft Copilot remain underrepresented in Thai-language applications, particularly within the pharmaceutical context, where empirical research is limited. To address this gap, this study aims to evaluate the performance of ChatGPT-4o, Google Gemini, and Microsoft Copilot in responding to drug-related queries in Thai language from the public.

## Methods

### Ethical Considerations

This study was approved by the Burapha University Ethical Committee (HS032/2567) with exemption from further review, as it involved publicly available data with no personal identifiers.

### Study Design and Duration

This was an analytical cross-sectional study, where data sources included public medical forums, whose websites were hosted by academic institutions (eg, the Faculty of Pharmacy’s websites) as well as social media. All questions were submitted freely by the public. The major data source was the Pantip website, the largest Thai online community since 1997. Although it is not a search engine, it offers a search function that enables the retrieval of relevant discussions across its forum categories. Its high traffic and diverse user demographic render it a valuable source for health-related queries. Additional data were obtained from public posts on Facebook and Twitter by using Thai hashtags such as #medicine, #herb, and #lab result (in Thai). Only publicly available content written in Thai was included. The data collection period referred to the posting dates of eligible questions, which spanned from November 1, 2019, through December 31, 2024.

### Query Retrieval and Selection Criteria

**Figure 1:**
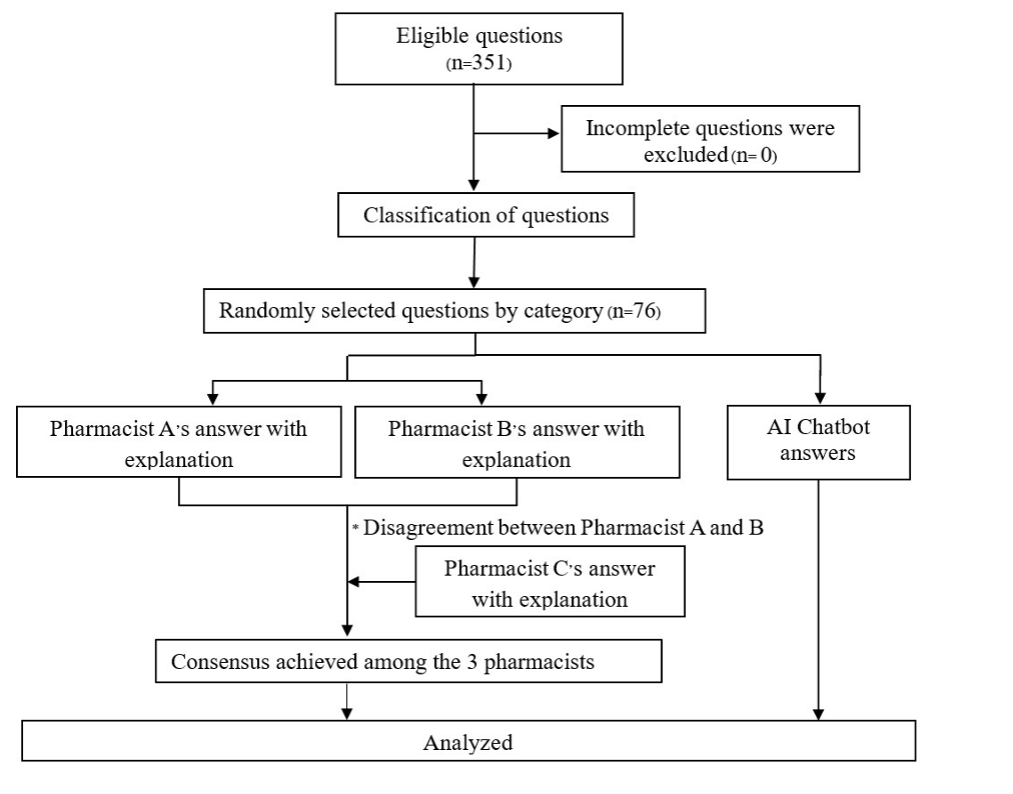
CONSORT (Consolidated Standards of Reporting Trials) diagram presenting the selection process of drug-related questions included in this study. AI: artificial intelligence.

The query selection process followed the CONSORT (Consolidated Standards of Reporting Trials) diagram (Figure 1). Three pharmacists independently retrieved drug-related questions from public databases in January 2025. The questions covered 18 predefined categories and an additional miscellaneous category for mixed-type questions. The 19 question categories and examples are presented in Multimedia Appendix 1. The drug-related question categories were classified based on systematic drug information response guidelines [14,15]. A minimum of 10 questions per category was selected, totaling at least 190 questions.

Duplicate checks and proper categorization were independently verified. Four questions per category were randomly selected using Microsoft Excel 2006. The pharmacists retrieved the answers from predefined reference sources for all questions before querying the AI models, preventing AI responses from biasing their evaluation. The selected questions were then submitted without modification to ChatGPT-4o [16], Google Gemini [17], and Microsoft Copilot [18] on March 1, 2025. ChatGPT responses were generated using the GPT-4o model via the ChatGPT Plus platform, accessed on March 1, 2025. Each question was submitted in a separate chat and analyzed within a single session, with all AI queries conducted on the same day to minimize potential variability from prior context or system updates.

The correctness, completeness, risk, and reproducibility of the responses were assessed by clinical pharmacists using predefined criteria. To obtain the correct answers, drug information was systematically collected from tertiary resources, including Micromedex [19], Lexicomp, Drug Interaction Facts [20], Drugs in Pregnancy and Lactation [21], Handbook on Injectable Drugs [22], MIMS Thailand [23], The Thai Food and Drug Administration [24], Summary of Product Characteristics, DiPiro’s Pharmacotherapy: A Pathophysiologic Approach, 12th edition [25], and both international and local clinical practice guidelines. Additionally, Thai herbal medicine databases, manufacturer catalogs, and distributor information were accessed to obtain local data. If data were incomplete, secondary resources (PubMed, ScienceDirect, Scopus) and primary resources were explored. Google Scholar was used as a supplementary tool with a reliability assessment. Examples of responses provided by the 3 AI systems compared with those of the pharmacists are presented in Multimedia Appendix 2.

### Evaluation of Correctness, Completeness, Risk, and Reproducibility Domains

The evaluation focused on 3 key domains: correctness, completeness, and risk, as assessed by the pharmacists. Reproducibility was tested by re-querying 1 random question per category after 1, 7, and 14 days. Two of the 3 pharmacists independently handled question retrieval, answer searching, and response evaluation. Disagreements were resolved by consensus or third-party review, as shown in Table 1.

**Table 1:** Evaluation of the 3 domains.

Key domains, subdomains			Categories and definitions
**Correctness**			
	Fully correct	The response is entirely accurate (2 points)	
	Partially correct	The response contains some inaccuracies (1 point)	
	Incorrect	The response is incorrect or does not address the core issue of the question (0 point)	
**Completeness**			
	Complete	The response addresses all aspects of the question comprehensively (2 points)	
	Partially complete	The response addresses some aspects of the question but omits others (1 point)	
	Incomplete	The response does not address the question and is irrelevant to the topic (0 point)	
**Risk**			
	No risk	The response is entirely accurate and complete, posing no harm to the patient if complied with as recommended (2 points)	
	Low risk	The response poses minimal harm but may contain inaccurate or incomplete information (1 point)	
	High risk	The response poses a significant risk to patient safety, such as the potential for adverse drug reactions, hospitalization, or delayed treatment if the advice is followed (0 point)	

### Statistical Analysis

The sample size was determined using α=.10 and power=.80. A prior study evaluating the suitability of ChatGPT for answering drug-related questions found that only 26% of the responses were satisfactory, while 74% were unsatisfactory due to inaccuracies, incomplete answers, or failure to address the question directly [26]. Therefore, at least 53 questions are required to achieve a 90% confidence level with an acceptable margin of error of 10% [27]. For content validity and balanced domain coverage, the final questions consisted of 76 items, which were structured into 19 prespecified categories with 4 items per category (19×4). The final item set was fixed before model evaluation. All statistical analyses were conducted using SPSS software (version 28.0; IBM Corp). The differences in the correctness, completeness, and risk were analyzed using a linear regression model with repeated measures. The analysis of reproducibility was performed using the coefficient of variation stated in the Association of Official Analytical Chemists Official Methods of Analysis [28].

## Results

### Overall Performance of ChatGPT-4o, Google Gemini, and Microsoft Copilot

This study retrieved 76 public questions from medical websites (36/76, 47%) and social media platforms, including Pantip (35/76, 46%), Facebook (1/76, 1%), and Twitter (4/76, 5%), posting from November 1, 2019, through December 31, 2024, and then submitted to ChatGPT-4o, Google Gemini, and Microsoft Copilot on March 1, 2025. The overall evaluation across all the question categories is illustrated in Table S1 of Multimedia Appendix 3.

Statistical analysis revealed that the type of AI chatbot did not significantly impact correctness, completeness, and risk (*P*=.08, *.*08, and .12, respectively). In contrast, the type of drug-related question significantly influenced the performance outcomes in correctness (*P*=.002) and risk (*P*=.04) but not in completeness (*P*=.23) (Multimedia Appendix 3). Although the differences in the correctness did not reach statistical significance (*P*=.08), a practical trend was observed. ChatGPT-4o demonstrated the highest tendency to provide fully correct responses and complete answers by addressing all the key points, followed by Google Gemini and Microsoft Copilot, respectively. For example, in adverse drug reaction–related questions on how to manage initial symptoms, ChatGPT-4o provided comprehensive and clinically useful answers that covered underlying causes, self-care strategies, symptom monitoring, and red-flag signs warranting medical attention. However, the other 2 systems offered only partially correct responses that lacked sufficient detail for practical application. Notably, Microsoft Copilot was the only AI system that provided entirely incomplete answers, primarily by generating content irrelevant to the question.

### Performance Correctness, Completeness, Risk, and Reproducibility of the AI Chatbots by Question Category

**Figure 2:**
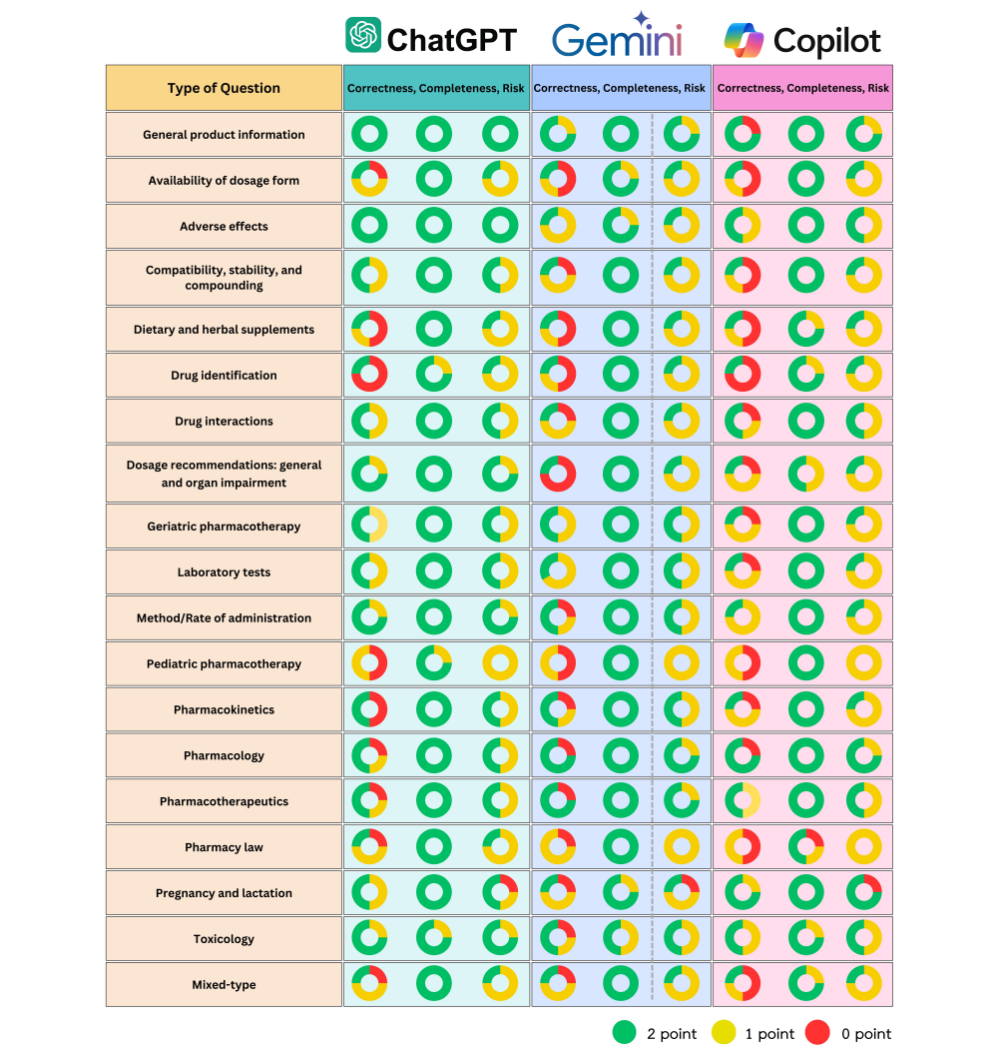
Performance of the artificial intelligence chatbots by question categories across the dimensions of correctness, completeness, and risk. One-fourth of the pie chart represents one question within each question category. Green, yellow, and red segments indicate a score of 2, 1, and 0, respectively.

The highest correctness ratings were observed in the general product information category, as shown in Figure 2. In this and the adverse effect category, ChatGPT provided fully correct responses to all the questions, while Google Gemini and Microsoft Copilot, respectively, yielded a lower rate of correctness. In contrast, Google Gemini and Microsoft Copilot demonstrated superior performance in pharmacology-related questions to ChatGPT. However, all 3 AI systems performed poorly in the following categories: pediatric pharmacotherapy, pharmacy law, availability of dosage forms, dietary and herbal supplements, drug identification, and mixed-type questions. Regarding online source citations, Microsoft Copilot used Drugs.com, NHS, WebMD, Medthai, Pantip, Mahidol University Faculty of Pharmacy, and PobPad websites as references, whereas ChatGPT and Google Gemini did not provide citations.

For completeness, ChatGPT and Google Gemini did not produce any incomplete responses for all the questions. In contrast, Microsoft Copilot yielded the highest number of partially complete responses and produced incomplete responses in the laboratory tests and pharmacy law categories. In terms of risk, all 3 AI chatbots produced high-risk responses. ChatGPT produced the highest responses categorized as no risk, followed by Microsoft Copilot and Google Gemini. Additionally, ChatGPT yielded a lower frequency of responses with lower risk than the other AI systems. Responses to pregnancy and lactation-related questions posed high risks across all 3 AI chatbots, indicating potential safety concerns.

In addition, there were no significant differences in performance across the correctness, completeness, or risk dimensions between the responses on days 1, 7, and 14. The responses to all the questions were unchanged across all 3 AI systems, with the coefficient of variation of less than 2% in all questions, demonstrating the reproducibility of the AI chatbots' responses.

## Discussion

### Principal Findings

This study provides new evidence on the performance of ChatGPT-4o, Google Gemini, and Microsoft Copilot in responding to drug-related queries in Thai. Overall, ChatGPT-4o achieved the highest proportion of fully correct and complete responses. This shows the improvement in the performance of ChatGPT since the 3.5 version provides incorrect answers in general [29]. In addition, the earlier version of ChatGPT provided inaccurate answers in the following domains: clinical guidelines, dosage adjustment, therapeutics, compounding and formulation, dosage regimens, compatibility and stability, adverse drug reactions, administration routes, drug-drug interactions, legal matters, foreign drug identification, toxicology, and multidomain queries [26,29-31]. A previous study also showed that the earlier version of ChatGPT had higher accuracy than Gemini [32]. However, the improved performance is not homogeneous across all question categories. We found that Gemini and Copilot performed better in specific areas such as pharmacology. Evidence availability may shape relative model performance because certain queries (eg, the combined use of central nervous system stimulants and antipsychotics) required theoretical reasoning in the absence of robust empirical evidence. This aligns with prior evidence showing that Gemini achieved higher concordance with clinical practice guidelines in evidence-based health advice than either Copilot or ChatGPT [33]. These limitations, particularly evident in Thai-language responses, indicate that despite advancements from version 3.5 to 4o, large language models still exhibit substantial limitations across various question categories.

Across all AIs, high-risk responses were rare but most evident in the pregnancy and lactation category. This highlights the limitations of generative AI models when responding to inquiries that rely on nuances. All 3 AI systems failed to recommend contraception during the use of medication, with clear evidence on the concurrent use of effective contraceptive methods. Such an omission could cause potential fetal harm. This observation is consistent with previous evidence [34], which found that ChatGPT-3.5 provided acceptable pregnancy-related responses in only 50% of the cases. Overall, our findings support the conclusion that the newer AI models, particularly ChatGPT-4o, have achieved improved accuracy and safety in responding to medication-related questions. Our study also shows that all 3 evaluated AI systems demonstrate a high degree of reproducibility. This shows that AIs have been improved since the previous versions of ChatGPT had reproducibility issues [29,30]. However, questions initially answered incorrectly continued to yield incorrect responses upon reassessment.

Regarding the language in evaluation, the earlier version of ChatGPT produces more accurate answers in response to English questions than other languages (eg, Turkish [5], Chinese [4]). The accuracy of ChatGPT in answering Chinese and Turkish medical questions was approximately 50% [4,5], agreeing with the accuracy of ChatGPT in answering Thai questions at 50%. The observed inaccuracies were attributed to several key factors. First, lexical and linguistic challenges such as informal vocabulary, abbreviations, slang, or transliterated English terms may lead to misinterpretation and incorrect responses [35], which could have serious consequences, particularly in patients with known drug allergies [36]. Second, variations in the underlying data sources used by each AI model influenced the quality of the responses. For instance, in this study, Microsoft Copilot often relied on nonmedical Thai sources, whereas ChatGPT and Google Gemini did not disclose their data sources. This raises concerns about transparency and source reliability. Third, the capacity of each AI system to handle complex cognitive tasks varied considerably. In this study, examples of such tasks included identifying locally manufactured drug products from text or image inputs, recognizing currently marketed dosage forms, interpreting legal implications of pharmacy-related scenarios, evaluating evidence levels between animal and human studies, and assessing the currency and clinical relevance of the information input. In addition, some of these domains (eg, pediatric pharmacotherapy, herbal and dietary supplements, locally marketed dosage forms) are particularly underrepresented in structured Thai resources such as Thai medical databases or drug labeling. This scarcity likely compounds the linguistic and contextual challenges described above, further limiting model accuracy in these categories.

This study offers valuable insights into the capabilities and limitations of AI chatbots in retrieving drug-related information in the Thai language. By evaluating responses from ChatGPT-4o, Google Gemini, and Microsoft Copilot across multiple question categories, we observed that AI systems were generally effective in addressing basic inquiries, particularly those involving general product information. These results support the potential utility of AI as a supplementary tool in routine information retrieval tasks, especially when queries are framed using clear formal language and include specific drug names. An additional strength of this study lies in the diversity of the question sources. All queries were collected from medical forums and social media platforms. This mix of sources introduced heterogeneity in terminology—from formal medical vocabulary to colloquial slang as well as variability in depth and clinical relevance. The diverse health literacy of the enquirers reflects the real-world patterns of medication information-seeking behavior [37]. However, the findings also underscore several critical limitations. The performance of all AI systems was markedly lower when addressing more complex or context-dependent domains such as pediatric pharmacotherapy, drug use in pregnancy and lactation, pharmacy law, and drug identification based on physical descriptions or images. In such cases, relying solely on AI systems may result in clinically significant inaccuracies, reinforcing the necessity for consultation with licensed health care professionals.

### Limitations

From a methodological perspective, this study was underpowered by the limited number of questions per category, which may have limited the detection of subtle differences in chatbot performance across subdomains. Expanding the sample size in future research would likely yield more insights. Additionally, this study primarily evaluates AI responses to general questions. Future investigations should assess AI performance in handling more individualized clinical scenarios to evaluate practical applicability and safety in real-world decision-making contexts. Moreover, because the study focuses exclusively on Thai queries, the findings may not be directly generalizable to other languages. Replication across diverse linguistic and clinical settings will be necessary to confirm the accuracy and reliability of AI systems.

### Conclusions

This study shows no statistically significant differences in correctness, completeness, or risk levels among ChatGPT-4o, Google Gemini, and Microsoft Copilot in responding to Thai-language drug-related questions. ChatGPT-4o yielded the highest number of fully correct and complete responses overall. However, Google Gemini and Microsoft Copilot performed relatively better for questions in pharmacology. All AI systems demonstrated notable limitations in addressing complex queries, including pediatric pharmacotherapy, pharmacy law, dosage form availability, dietary and herbal supplements, drug identification, pregnancy and lactation, and mixed-type drug inquiries. Although response risks were observed in all systems, ChatGPT-4o produced the highest proportion of no-risk responses. The reproducibility of the 3 AIs was consistent, though incorrect responses remained unchanged when reassessed.

## Appendix

Multimedia Appendix 1 Sample questions by medication category.

Multimedia Appendix 2 Examples of responses provided by artificial intelligence compared to those given by a pharmacist.

Multimedia Appendix 3 Assessment of the overall performance of ChatGPT-4o, Google Gemini, and Microsoft Copilot across the dimensions of correctness, completeness, and risk.

Multimedia Appendix 4 CONSORT-eHEALTH checklist (V 1.6.1).

## Abbreviations

AI: artificial intelligence
CONSORT: Consolidated Standards of Reporting Trials

## Data Availability

All data generated or analyzed during this study are included in this manuscript and in the appendices.
