# Supplementary material for: Effectiveness of ChatGPT, Google Gemini, and Microsoft Copilot in Answering Thai Drug Information Queries: Cross-Sectional Study
Source: JMIR AI. 2025 Dec 15;4:e79751. doi: 10.2196/79751 (PMC12750067; doi:10.2196/79751)
Supplement: Multimedia Appendix 1 [file ai_v4i1e79751_app1.docx]

Multimedia Appendix 1: Sample Questions by Medication Category

| **No.** | **Categorized questions** | **Questions** |
| --- | --- | --- |
| 1 | General product information | Do Phet Sangkhayat and Daflon contain steroids? |
| 2 | Availability of dosage form | Is Molnupiravir 200 mg for COVID-19 available in pharmacies in Thailand? |
| 3 | Adverse effects | I’m a 17-year-old transgender woman taking daily hormonal birth control (Prim). Can I get the COVID-19 vaccine, and what side effects might I have? |
| 4 | Compatibility, stability, and compounding | I used antibiotic eye drops for a stye. The symptoms are gone—how long can I keep the drops in the fridge before they expire? |
| 5 | Dietary and herbal supplements | I'm taking antihypertensive drugs. Can I also take Thao Wan Priang (a Thai herbal remedy)? Will it interfere with my blood pressure meds? |
| 6 | Drug identification | Are Fucidin (orange stripe) and Fucidin H (red-black stripe) interchangeable? The pharmacist said they are, but Fucidin H may be gentler. I'm worried it might make my wound worse. |
| 7 | Drug interactions | Can I take Ciprofloxacin 500 mg and Metronidazole 400 mg at the same time? |
| 8 | Dosage recommendations: general and organ impairment | My doctor prescribed Amoxy 500 mg, 2 times a day for 20 days (40 capsules). Is this a normal course? |
| 9 | Method/Rate of administration | A 12-year-old child weighing 50 kg cannot swallow Augmentin 1g tablets. Can we crush the tablet or switch to the liquid form? |
| 10 | Pharmacotherapeutics | I took Amoxicillin 500 mg for a cold (sore throat, cough, runny nose), but later found out it might not help. I’ve only taken 1 tablet—can I stop now? |
| 111 | Geriatric pharmacotherapy | My grandfather, who is 89 years old, has memory problems. Are there any medications suitable for elderly people with this issue? |
| 12 | Pediatric pharmacotherapy | Can an 11-year-old girl who already has her period take medicine to delay menstruation? |
| 13 | Pharmacokinetics | I've been constipated for 4 days. I’ve taken Xenolac for 2 days (2 tablets on day 1, 4 tabs on day 2) but no effect. How long does this medicine take to work? |
| 14 | Laboratory tests | My HIV test was negative, but syphilis was “reactive.” What could cause that? |
| 15 | Pharmacology | Does Albendazole kill both tapeworms and their eggs before they are passed out with stool? |
| 16 | Pharmacy law | Where can I buy “Ta-Dol green-yellow”? The pharmacy said they won’t sell it. |
| 17 | Pregnancy and lactation | Can Rybelsus be used during pregnancy? Should it be stopped before trying to conceive? |
| 18 | Toxicology | I overdosed on Tramadol HCl 50 mg. I feel dizzy, high like from cannabis, with a headache but no nausea. What should I do? |
| 19 | A mixed-type drug question | I take Parlodel 2.5 mg (1 tablet, morning and evening). If I want to take Actifed, how long should I stop Parlodel before? Taking them together makes my nasal congestion worse. I also tried CPM and Loratadine, but they didn’t help. What can I take that won’t interact? |
