# Supplementary material for: Effectiveness of ChatGPT, Google Gemini, and Microsoft Copilot in Answering Thai Drug Information Queries: Cross-Sectional Study
Source: JMIR AI. 2025 Dec 15;4:e79751. doi: 10.2196/79751 (PMC12750067; doi:10.2196/79751)
Supplement: Multimedia Appendix 2 [file ai_v4i1e79751_app2.docx]

Multimedia Appendix 2: Examples of responses provided by AI compared to those given by a pharmacist

| **Question** | **ChatGPT-4o** | **Google Gemini** | **Microsoft Copilot** | **Pharmacists** |
| --- | --- | --- | --- | --- |
| Do I need to store Hista-Op (yellow cap) eye drops in the refrigerator?  As the title says, I’m currently being treated for pterygium, and my doctor prescribed this eye drop. The staff at the pharmacy counter told me to keep it in the fridge. But when I searched online, I found mixed information. So, I’d like to ask—does this brand of eye drop really need to be refrigerated, or is it okay to store it at room temperature?  Also, if it must be refrigerated all the time, what should I do when I go to school since I can’t store it in the fridge there? | Hista-Op (Ketotifen 0.025%) is an eye drop used to treat allergic eye symptoms such as itching, redness, and pterygium. It belongs to the antihistamine group.  Storage instructions:  It can be stored at room temperature (not exceeding 25°C). If the weather is very hot, it’s better to keep it in the refrigerator (but not in the freezer). Keep it away from direct sunlight and heat.  It’s safe to carry it in a regular bag if you avoid hot places like near windows or inside a car. If your classroom is very warm, you can use a small cooling pouch (ice is not necessary).  Recommendation:  If you need to use it frequently and are concerned about heat, it’s a good idea to ask your pharmacist for advice. You might also consider bringing only the amount needed for the day instead of carrying the whole bottle. | In general, Hista-Oph (yellow cap) eye drops do not need to be stored in the refrigerator. They can be kept at room temperature, but should be kept away from direct sunlight and heat. However, here are some important storage tips:  Before opening: If the bottle is still sealed, you may store it in a regular refrigerator (2–8°C) to help extend its shelf life.  After opening: Once opened, there’s no need to refrigerate. You can keep it at room temperature, but make sure to use it within one month.  Storage: Keep the bottle tightly closed, avoid sunlight and heat, and store it out of reach of children.  Taking it to school: It’s okay to carry the drops in your bag. Just avoid placing it in very hot areas, like a car parked in the sun.  Additional tips:  If you’re unsure, it’s best to ask the doctor or pharmacist who prescribed the medication. Also, check the drops regularly—if the color or appearance changes, do not continue using them.  Hope this information is helpful! Feel free to ask if you have more questions. | Hista-OP eye drops (yellow cap) should be stored in a regular refrigerator (around 4°C) to maintain the drug’s effectiveness and help prevent bacterial growth.  However, if you need to take the drops to school and can't keep them refrigerated all the time, you can carry them in an insulated bag or a portable medicine case with a cold gel pack to help maintain a cool temperature.  If you have any further questions, it’s best to consult your doctor or pharmacist to ensure safe and proper use of the medication. | Hista-Oph does not have a yellow cap—it has a green cap.  Its active ingredients are Tetrahydrozoline and Antazoline, and it does not need to be refrigerated. It can be stored at room temperature, but should not be left in a car. It’s safe to carry with you to school.  However, eye drops with a yellow cap sold in Thailand may refer to different products:  Chlor-Oph (yellow cap): contains Chloramphenicol, an antibiotic that must be refrigerated.  Dex-Oph (also yellow cap): contains a combination of a steroid and an antibiotic—this may also need cold storage depending on the formulation.  Lac-Oph: an artificial tear solution with a yellow cap, does not require refrigeration.  So, it’s important to read the label carefully to confirm both the generic name and the brand name before deciding how to store the eye drops. |
