# Supplementary material for: Effectiveness of ChatGPT, Google Gemini, and Microsoft Copilot in Answering Thai Drug Information Queries: Cross-Sectional Study
Source: JMIR AI. 2025 Dec 15;4:e79751. doi: 10.2196/79751 (PMC12750067; doi:10.2196/79751)

**Multimedia Appendix 3.**

Table S1. Table of Correctness, Completeness, and Risk result

|  | ChatGPT (n = 76) | Gemini (n = 76) | Copilot (n = 76) |
| --- | --- | --- | --- |
| Correctness |  |  |  |
| Fully | n = 38 (50.00%,  95% CI: 38.30, 61.70) | n = 26 (34.21%,  95% CI: 23.71, 45.99) | n = 27 (35.53%,  95% CI: 24.88, 47.34) |
| Partial | n = 24 (31.58%,  95% CI: 21.39, 43.26) | n = 29 (38.16%,  95% CI: 27.25, 50.02) | n = 27 (35.53%,  95% CI: 24.88, 47.34) |
| Incorrect | n = 14 (18.42%,  95% CI: 10.45, 28.97) | n = 21 (27.63%,  95% CI: 17.99, 39.09) | n = 22 (28.95%,  95% CI: 19.11, 40.48) |
| Completeness |  |  |  |
| Fully | n = 73 (96.05%,  95% CI: 88.89, 99.18) | n = 71 (93.42%,  95% CI: 85.31, 97.83) | n = 67 (88.16%,  95% CI: 78.71, 94.44) |
| Partial | n = 3 (3.94%,  95% CI: 0.82, 11.11) | n = 5 (6.58%,  95% CI: 2.17, 14.96) | n = 7 (9.21%,  95% CI: 3.78, 18.06) |
| Incorrect | n = 0 (0.00%,  95% CI: 0.00, 4.74) | n = 0 (0.00%,  95% CI: 0.00, 4.74) | n = 2 (2.63%,  95% CI: 0.32, 9.18) |
| Risk |  |  |  |
| No | n = 38 (50.00%,  95% CI: 38.30, 61.70) | n = 26 (34.21%,  95% CI: 23.71, 45.99) | n = 27 (35.53%,  95% CI: 24.88, 47.34) |
| Low | n = 37 (48.68%,  95% CI: 37.04, 60.43) | n = 49 (64.47%,  95% CI: 52.66, 75.12) | n = 48 (63.16%,  95% CI: 51.31, 73.94) |
| High | n = 1 (1.32%,  95% CI: 0.03, 7.11) | n = 1 (1.32%,  95% CI: 0.03, 7.11) | n = 1 (1.32%,  95% CI: 0.03, 7.11) |

Figure S1. Percentage-based assessment of the overall performance of ChatGPT-4o, Google Gemini, and Microsoft Copilot across the dimensions of correctness, completeness, and risk.


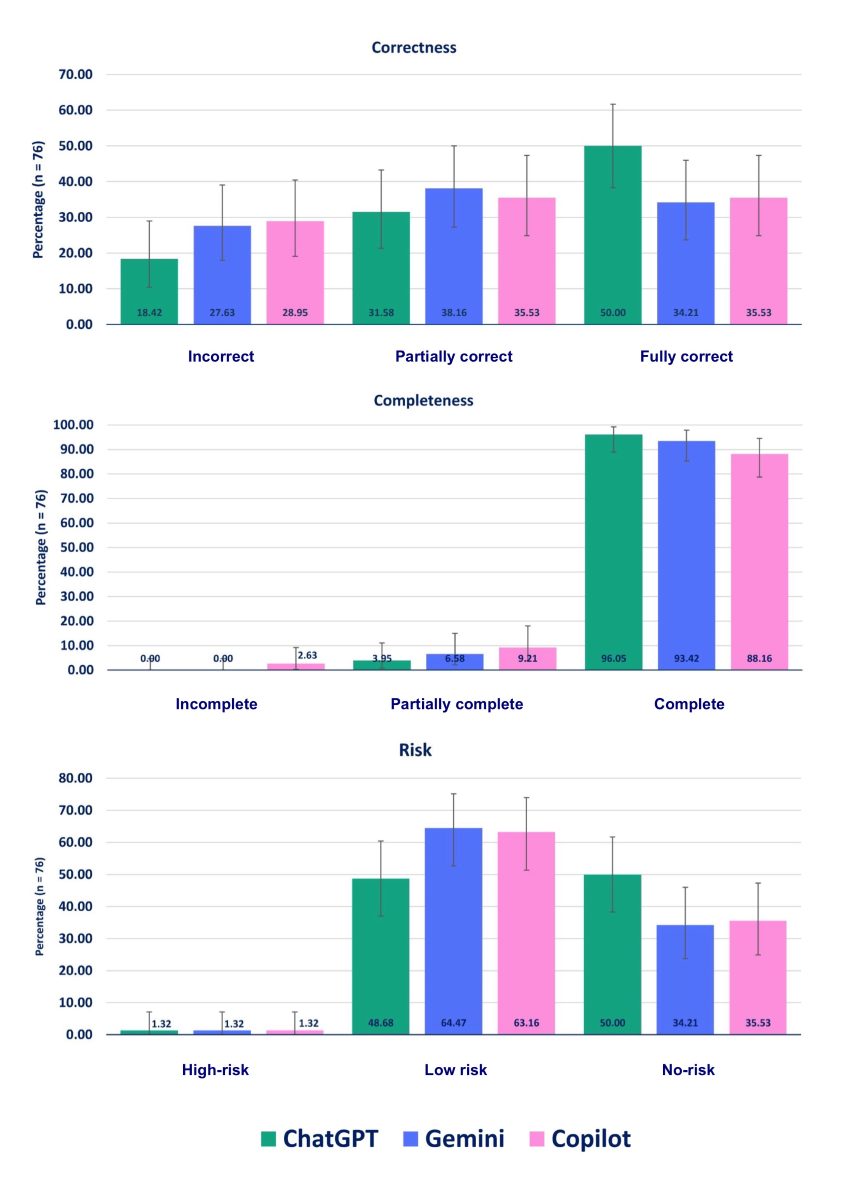

Supplement: Multimedia Appendix 3 [file ai_v4i1e79751_app3.docx]
